# Inhibition of Photoconversion Activity in Self-Assembled ZnO-Graphene Quantum Dots Aggregated by 4-Aminophenol Used as a Linker

**DOI:** 10.3390/molecules25122802

**Published:** 2020-06-17

**Authors:** Kyu Seung Lee, Young Jae Park, Jaeho Shim, Guh-Hwan Lim, Sang-Youp Yim, Jin Won Seo, Jae Hyoung Ryu, Dong Ick Son

**Affiliations:** 1 Institute of Advanced Composite Materials, Korea Institute of Science and Technology, 92, Chudong-ro, Bongdong-eup, Wanju-gun, Jeollabuk-do 55324, Korea; kslee4268@gmail.com (K.S.L.); jshim0214@gmail.com (J.S.); gunanwow@gmail.com (G.-H.L.); 2 Light Convergence Research Team, Korea Institute of Lighting and ICT, 370, Dongseo-ro, Iksan-si, Jeollabuk-do 54630, Korea; yjpark@kilt.re.kr (Y.J.P.); phynux@kilt.re.kr (J.H.R.); 3 Advanced Photonics Research Institute, Gwangju Institute of Science and Technology, 123, Cheomdangwagi-ro, Buk-gu, Gwangju 61005, Korea; syim@gist.ac.kr; 4 Department of Materials Engineering, KU Leuven, 3001 Leuven, Belgium; maria.seo@kuleuven.be; 5 KIST School, Department of Nanomaterials and Nano Science, University of Science and Technology (UST), 217, Gajeong-ro, Yuseong-gu, Daejeon 34113, Korea

**Keywords:** ZnO-graphene core-shell QDs, aggregates, charge separation, photocatalyst, photodetector, solar energy conversion

## Abstract

The aggregation of zinc oxide nanoparticles leads to an increased absorbance in the ultraviolet-visible region by an induced light scattering effect. Herein, we demonstrate the inhibition of photoconversion activity in ZnO-graphene core-shell quantum dots (QD) (ZGQDs) agglomerated by 4-aminophenol (4-AP) used as a linker. The ZnO-graphene quantum dots (QD) aggregates (ZGAs) were synthesized using a facile solvothermal process. The ZGAs revealed an increased absorbance in the wavelengths between 350 and 750 nm as compared with the ZGQDs. Against expectation, the calculated average photoluminescence lifetime of ZGAs was 7.37 ns, which was 4.65 ns longer than that of ZGQDs and was mainly due to the high contribution of a slow (τ_2_, τ_3_) component by trapped carriers in the functional groups of graphene shells and 4-AP. The photoelectrochemical (PEC) cells and photodetectors (PDs) were fabricated to investigate the influence of ZGAs on the photoconversion activity. The photocurrent density of PEC cells with ZGAs was obtained as 0.04 mA/cm^2^ at 0.6 V, which was approximately 3.25 times lower than that of the ZGQDs. The rate constant value of the photodegradation value of rhodamine B was also decreased by around 1.4 times. Furthermore, the photoresponsivity of the PDs with ZGAs (1.54 μA·mW^−1^) was about 2.5 times as low as that of the PDs with ZGQDs (3.85 μA·mW^−1^). Consequently, it suggests that the device performances could be degraded by the inhibition phenomenon of the photoconversion activity in the ZGAs due to an increase of trap sites.

## 1. Introduction

Zinc oxide nanoparticles (ZnO NPs) are known to be environmentally friendly substances, which have an excellent photo conversion activity, good photo responsibility in the UV-visible range, and unique physical properties. Owing to these outstanding properties, they have been applied to photodetectors [1,2,3,4], solar cells [5,6,7], and photocatalysts [8]. In tandem with this, many researchers have reported several approaches to improve the photoelectric conversion efficiency through the structural modification of ZnO NPs [9]. In particular, the interconnected structure was formed by increasing the size of ZnO NPs through heat treatment, which showed better photoelectric conductivity [10]. Nevertheless, the large-sized ZnO NPs are limited to improving photo conversion activity due to relatively low light scattering resulting from reduced internal surface area. Therefore, ZnO NP aggregates have been investigated for effective photon acquisition by strong light scattering as an alternative method [10,11]. However, ZnO has low photostability due to photocorrosion, which hinders the photoconversion activity [12,13]. To suppress the photocorrosion of ZnO NPs, Kim et al. proposed a conformal coating of graphene and C_60_ on ZnO NPs. The core-shell ZnO-nano carbon hybrid structures provided an effect of photocorrosion prevention, as well as an improved charge transport capability of ZnO NPs [14]. However, the ZnO-nano carbon hybrid composites had oxygenated functional groups on their surfaces such as epoxy, hydroxyl, and carboxyl groups that acted as trap sites of photoelectrons, which led to a degradation of the device performance. Many approaches to forming ZnO aggregates have used the method of increasing the size of ZnO itself [10,11]. Conversely, using our approach it is possible to form aggregates of ZnO quantum dots which are individually graphene wrapped. In this paper, we demonstrate the synthesis of the zinc oxide graphene quantum dot aggregates (ZGAs) using 4-aminophenol (4-AP) as a linker by a facile solvothermal method. The arylamine derivatives including 4-AP are employed for the covalent functionalization of graphene [15], which can play a role as a medium that can connect between the graphene shells of ZnO-graphene core-shell quantum dots (ZGQDs). Herein, we investigated the photoconversion properties of ZGAs and ZGQDs using the UV-Vis spectrophotometer, and time-resolved photoluminescence (TRPL). The structural properties were analyzed using scanning electron microscopy (SEM), transmission electron microscopy (TEM), X-ray diffraction (XRD), Raman spectroscopy, and Fourier-transform infrared (FT-IR) spectroscopy. We also fabricated the ZGA- and ZGQD-based photodetectors (PDs), and a photoelectrochemical (PEC) cell to demonstrate the role of the suggested materials.

## 2. Results and Discussion

**Figure 1:**
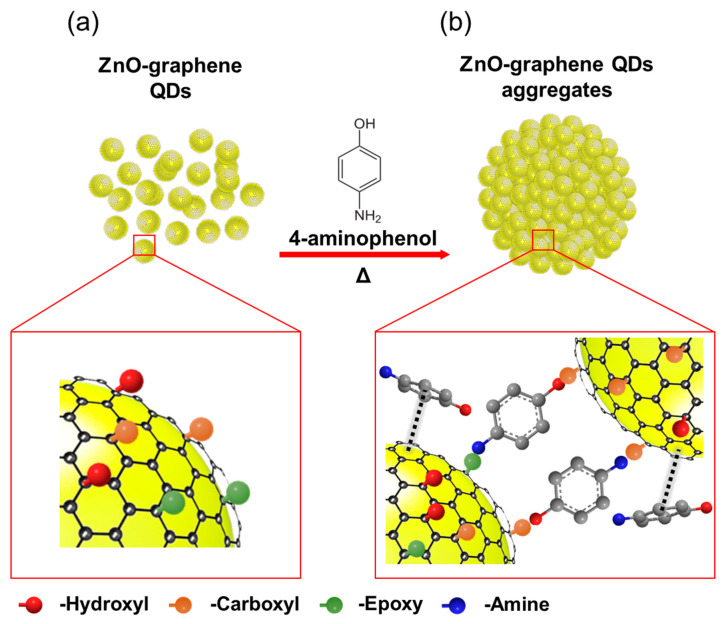
Schematic of the zinc oxide graphene quantum dot aggregates (ZGAs) synthesis process. (**a**) The ZnO-graphene core-shell quantum dots (ZGQDs) were aggregate by 4-AP; (**b**) The micro-sized ZGQDs aggregates.

**Figure 2:**
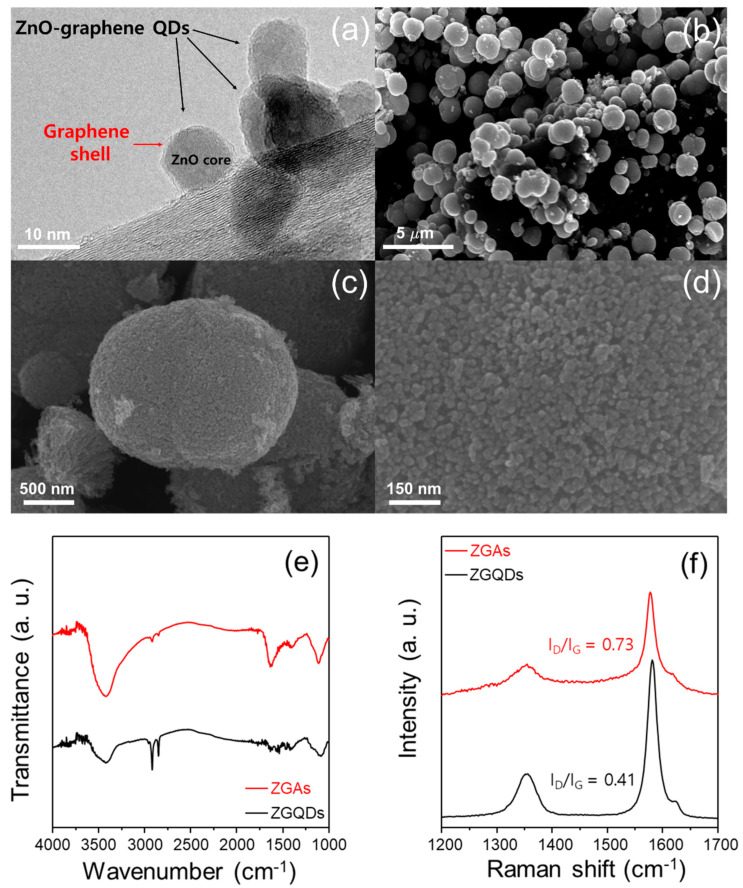
(**a**) High-resolution transmission electron microscopy (HR-TEM) image of ZGQDs; (**b**,**c**) Scanning electron microscopy (SEM) image of ZGAs; (**d**) SEM image of densely packed ZGQDs; (**e**) Fourier-transform infrared (FT-IR) spectra of ZGAs and ZGQDs; (**f**) Raman spectra of ZGAs and ZGQDs with a laser source of 514 nm.

The schematic diagram for the synthesis of the ZGAs is shown in Figure 1. Figure 1a depicts the ZGQDs chemically synthesized in dimethylformamide (DMF) solution, which have three kinds of functional groups on the surface of graphene [16]. The aggregation mechanism of ZGAs can be described by several chemical reactions as follows (Figure 1b): (1) the activated carboxyl groups of ZGQDs react with the amine groups of 4-AP to form amide bonds at a high temperature of 140 °C [15], (2) the hydroxyl groups remaining in 4-AP form ester bonds with the carboxyl groups of another graphene surface by the esterification reaction, (3) the amine groups of 4-AP react with epoxy groups on the graphene nanoshell through the ring-opening reaction [17], and (4) the noncovalent interaction (pi-interaction) between graphene shells of ZGQDs and aromatic molecules of 4-AP [18]. Consequently, the graphene shell serves as an important intermedium for bonding with other ZGQDs. We also attempted to synthesize ZnO aggregates without graphene shell in the same way. Figure S2 (in Supplementary Materials) showed randomly distributed irregular agglomeration. This result implies that the chemical bond between the oxygenated functional groups of graphene shells and 4-AP acts as a linker to connect each other. As these reactions occur continuously with the surrounding ZGQDs, the ZGQDs agglomerate together to form larger clusters. The structure of ZGAs could be changed depending on the concentration of 4-aminophenol and the reaction time. To closely investigate the size and morphology of the ZGQDs, TEM and SEM were employed. The HR-TEM image clearly shows that the graphene shell is formed outside the ZnO core, as shown in Figure 2a. The spherical ZGAs are uniformly formed with the lowest surface energy, as shown in Figure 2b,c. The size of ZGAs is distributed between 0.2 and 2.0 μm, and the dominant particle diameter is ∼1.5 μm, as shown in Figure S1 (in Supplementary Materials). Figure 2d shows the densely packed ZGQDs. To understand the formation process of aggregates from the chemical reaction, we employed the FT-IR measurements. Figure 2e shows the transmittance of ZGAs and ZGQDs as a function of the wavenumber. Four peaks are observed in ZGAs at 3400, 1615, 1400, and 1066 cm^−1^, which correspond to –COOH, C=C, C–H, and C–O–C stretching, respectively. The decreased intensities of the peaks located at 2850 and 2917 cm^−1^ are related to the C–H stretching and bending vibrations [19]. The relatively strong peak intensity at 1622 cm^−1^ as compared with ZGQDs can be attributed to the C–O–C stretching vibration, indicating successful esterification between ZGQDs and 4-AP. Furthermore, the observed peak at 1650 cm^−1^ corresponds to the amide bond (–CONH), which signifies that the ZGQDs is successfully agglomerated by the chemical interaction between 4-AP and ZGQD [20]. To investigate the successful synthesis and materials quality of ZGAs, micro-Raman spectroscopy with a 514 nm laser was employed. It has proven to be an essential tool for studying chemical bonding of carbon-based materials. The D band indicates defects and disturbances in chemically functional graphene sheets, and the G band corresponds to the E_2g_ mode of the sp^2^-bonded carbon atoms and the vibration mode of k-point phonons with A_1g_ symmetry in a two-dimensional hexagonal lattice (Figure 2f) [21,22]. The D bands of ZGAs and ZGQDs are observed at the same location of 1354 cm^−1^, while the G band of ZGAs is located at 1577 cm^−1^, which is downshifted by 3 cm^−1^ as compared with that of ZGQDs. The G band shift is associated with the chemically doped carbon materials [23,24]. The I_D_/I_G_ ratio of ZGAs and ZGQDs showed 0.73 and 0.41, respectively, which is mainly attributed to the covalent modification by esterification reaction between the sp^3^ carbons on the graphene shells and hydroxyl groups of 4-AP [25,26].

**Figure 3:**
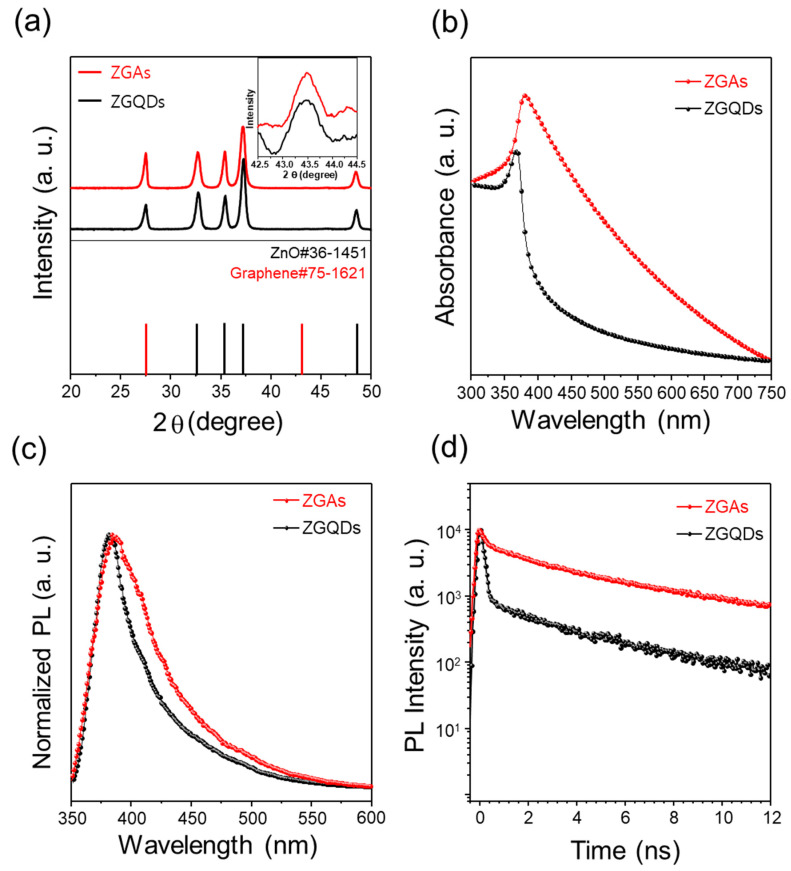
(**a**) X-ray diffraction (XRD) spectra of ZGAs and ZGQDs; (**b**) Absorbance spectra of ZGAs and ZGQDs; (**c**) Photoluminescence (PL) spectra of ZGAs and ZGQDs (excitation source: 350 nm); (**d**) Time-resolved photoluminescence (TRPL) spectra of ZGAs and ZGQDs.

Figure 3a shows the diffraction peaks of ZGAs and ZGQDs that are exhibited at 32.5°, 35.5°, 34.4° and 47.5° corresponding to the ZnO crystal planes of (100), (102), (101) and (102), respectively. Moreover, two additional peaks are also observed at 25.8° and 42.5° corresponding to the (002) and (100) planes for the graphene. The results are well matched with the reference patterns of ZnO (JCPDS no. 36-1451) and graphene (JCPDS no. 75-1621). In addition, the particle size was calculated using the following Scherrer equation based on the obtained diffraction patterns to confirm that ZGAs are comprised of ZGQDs. D = Κλ/βcosθ, where D is crystallite size (nm), Κ represents the Scherrer constant, λ is wavelength of the x-ray sources about 0.15406 nm, β is full width half maximum (FWHM), and θ is peak position. The average crystallite sizes of ZGQDs and ZGAs were calculated to be about 16.9 and 16.5 nm, respectively. These results indicate that ZGAs consist of numerous ZGQDs bound by aromatic linkers. The UV-Vis absorption spectra of ZGAs and ZGQDs were collected to characterize the optical properties as shown in Figure 3b. The optical absorption behavior is one of the important fundamental properties in revealing the energy structure of ZGQDs and ZGAs. The absorption spectra present that the excitonic peaks of ZGAs and ZGQDs are observed at 379 and 368 nm, respectively. The excitonic peak of ZGAs was red-shifted, which could be mainly due to the pi-interaction between the graphene shell of ZGQDs and 4-AP [18]. Compared with ZGQDs, the enhanced absorption of ZGAs at the wavelengths ranging from 290 to 345 nm can be originated from the 4-AP [27]. Furthermore, the absorption of ZGAs in the wavelengths between 350 to 725 nm increased due to an effective photon capture by a strong light scattering effect [28,29]. The enhanced absorption in the wide wavelength range plays a critical role in improving photoconversion efficiency [28,29]. The normalized PL spectra of ZGAs and ZGQDs were obtained at room temperature to investigate the optical properties, as shown in Figure 3c. The normalized PL spectra of ZGQDs reveals a ZnO-related peak at around 381 nm and a shoulder peak of 411 nm provoked by the graphene shell of ZGQDs, which are in good agreement with previous reports [16,25,30]. The ZnO-related peak of ZGA is located at 385 nm, which is redshifted by approximately 5 nm. The broadening of the PL emission in ZGAs results from the influence of chemical bonding through esterification, amination reactions, and pi-interaction between the graphene shells and 4-AP [18]. To further investigate the behavior of photoexcited electrons in ZGAs and ZGQDs, the TRPL was performed (Figure 3d). The 5 mg of ZGAs and ZGQDs powders were dispersed in 1 mL of ethanol. The sample solutions were treated by ultrasonication for 30 min, which were transferred to a quartz cell. The TRPL measurements were performed using a streak camera with an excitation source of ∼150 fs, 350 nm pulses derived by 2nd harmonic generation from an 80 MHz Ti:Sapphire laser with a wavelength of 700 nm. The temporal resolution was 40 ps. The PL lifetimes of ZGAs and ZGQDs were fitted by the tri-exponential function as follows: I(t) = A_1_exp(−t/τ_1_) + A_2_exp(−t/τ_2_) + A_3_exp(−t/τ_3_). The parameters (τ_1_, A_1_, τ_2_, A_2_, τ_3_, A_3_) derived from the analyses of TRPL data for ZGAs and ZGQDs are shown in Table S1 (in Supplementary Materials). The lifetimes in terms of fast (τ_1_) and slow (τ_2,_ τ_3_) components for ZGAs and ZGQDs are similar, but the proportion of fast component in the ZGQDs is 18% higher than that of the ZGAs. It can be explained by the fact that most of the excited electrons from the ZnO core fall down to the bottom of the conduction band without electron traps in the functional groups of 4-AP. However, relatively large numbers of the excited electrons in ZGAs could be captured in both the functional groups of the graphene shells and the 4-AP acted as an aromatic linker. As a consequence, the average lifetime (τ_avg_) of ZGAs increased to 7.37 ns, which was 4.63 ns slower than that of ZGQDs.

**Figure 4:**
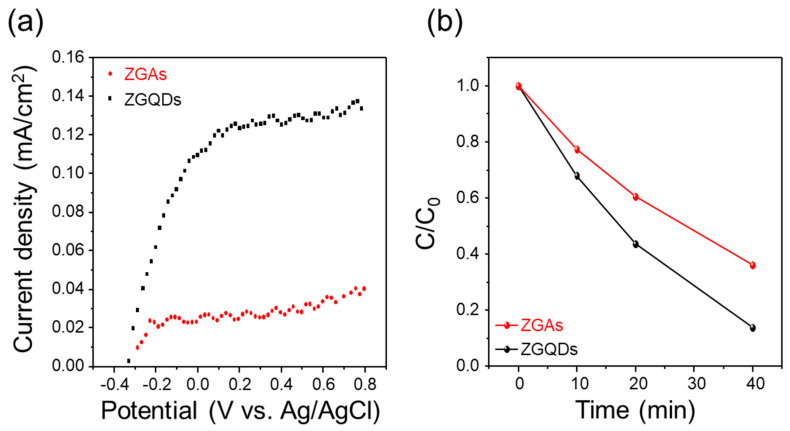
(**a**) Current density to potential (J-V) curves of ZGAs and ZGQDs; (**b**) Concentration changes of rhodamine B (Rh B) aqueous solution from initial concentration (C_0_) as a function of irradiation time.

In order to verify the photoconversion activity of ZGAs and ZGQDs, the PEC cells and the photodetectors based on ZGAs and ZGQDs were fabricated. Figure 4a shows the photocurrent densities of PEC cells based on ZGAs and ZGQDs measured under 1 sun illumination (AM 1.5 G, 100 mW/cm^2^), using a three electrode system in 0.5 M NaClO_4_ electrolyte (pH 6.9). The photoanode films of ZGAs and ZGQDs were prepared on FTO glass using a conventional doctor blade method. The photoactive area of PEC was around 5 by 5 mm. The photocurrent densities of ZGAs and ZGQDs were 0.04 mA/cm^2^ and 0.13 mA/cm^2^ at 0.6 V for ZGAs and ZGQDs, respectively. The photoexcited electrons can be rapidly transferred to LUMO levels of graphene shells, and the holes can participate in the oxidation process [14]. To confirm the photocatalytic activity of ZGAs and ZGQDs, the photodegradation experiment was performed using 0.01 mM rhodamine B (rhB) aqueous solution under 1 sun illumination. The prepared sample powders were dispersed into rh B aqueous solution. After 2 h stirring in dark conditions, then AM 1.5 G of light was irradiated and 1 mL of sample solution was extracted at intervals, and then centrifuged at 10,000 rpm. The concentration change of the obtained supernatant was observed through UV-Vis spectroscopy.

Figure 4b presents the rate constant (C/C_0_) as a function of time after 40 min illumination. The decomposition rates of rhB were 64% and 86% for the PEC cells with ZGAs and ZGQDs. In addition, the degradation reaction constant values were calculated to be 0.016 and 0.022 for the ZGAs and the ZGQDs using 1st order reaction [31]. These results are caused by oxidation with the photoinduced holes in valence band of ZGAs and ZGQDs [32].

**Figure 5:**
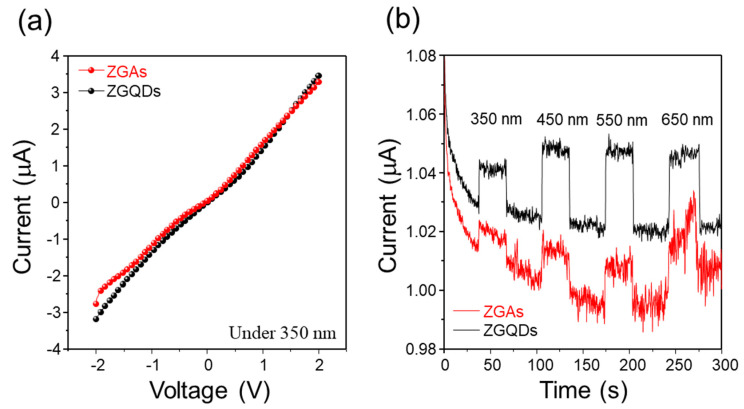
(**a**) Current-voltage (I-V) characteristics of photodetectors with ZGAs and ZGQDs under illumination with 350 nm; (**b**) Current-time (I-t) curves of the photodetectors with ZGAs and ZGQDs at 1 V under illumination with 350, 450, 550, and 650 nm sources.

In order to examine the effect of ZGAs and ZGQDs on photosensitivity in terms of various incident light sources, we fabricated the photodetectors based on ZGAs and ZGQDs with conventional metal-semiconductor-metal (MSM) structures. The ZGAs and ZGQDs were prepared dispersed in ethanol (5 mg/10 mL), and then drop casting on the Cr/Au (10 nm/100 nm) IDT patterned SiO_2_/Si substrate, followed by annealing at 110 °C for 10 min under ambient condition. The prepared MSM structures were electrical characterized by Keithely 4200 semiconductor properties tester with the optical sources using a 150 W Xenon lamp. Figure 5a shows the photocurrent as a function of applied bias under irradiation at 350 nm with a power density of 0.13 mW·cm^−2^ and the effective area of photodetector was 0.2 cm^2^_._ The current-voltage (I-V) curves under illumination states shows the ohmic behavior. The photocurrents of ZGAs and ZGQDs at 1 V were increased by around 0.5% and 1.4% as compared with those under dark state. The I-V curves were measured under dark condition, as shown in Figure S3 (in Supplementary Materials). Figure 5b presents the current-time (I-t) photoresponse at an applied voltage of 1 V under illumination states with various wavelengths. The ZGQD-based photodetector obviously shows the dynamic photoresponse, whereas the ZGA-based photodetector presents comparatively unstable photoresponses. To closely evaluate the photodetection performance of ZGA- and ZGQD-based photodetector, we summarized the performance of the photodetectors with ZGAs and ZGQDs (Table 1). The sensitivity was calculated from the following equation [33]: S = (J_p_ − J_d_)/J_d_, where J_p_ is current density under illumination state, and J_d_ is current density under dark state. In addition, the photo responsivities were calculated by the following equation [33]: R_λ_ = (I_p_ − I_d_)/*PA,* where I_p_ is the photocurrent, I_d_ is the dark current, P is the signal intensity, and A is the optical signal exposed area of photodetector. The calculated sensitivity and responsivity of the device with ZGAs were obtained as 0.005 and 1.54 μA·mW^−1^, which were about 2.5 times lower than that with ZGQDs. Taken together, the photoconversion devices with ZGAs exhibited lower efficiency as compared with those with ZGQDs, which can also be supported by the fact that τ_avg_ of ZGAs increased due to the carrier trap in the functional groups of the linker, as described above. The photoexcited electrons are transferred to graphene shells and adjacent ones via aromatic linker simultaneously. Then, plenty of the photoexcited electrons can be captured by oxygenated functional groups of the graphene shells, as well as the 4-AP acted as a linker. Consequently, the ZGAs agglomeratd by 4-AP can impede the photoconversion activity by decreased conductivity. Typically, metal oxide aggregates have improved photoconversion activity due to enhanced light absorption by strong light scattering. Unfortunately, the ZGAs have different results, which is thought to be due to charge trap issues. The results of TRPL supported the inhibition behavior of the photoconversion activity of ZGAs. To solve the inhibition of photoconversion activity, it is believed that the oxygenated functional groups on the graphene surface can be solved through additional chemical functionalization.

## 3. Experimental Details

### 3.1. Synthesis of ZGAs

The 5 g of graphite powder was mixed with 72 mL of the 3:1 mixture of sulfuric acid and nitric acid. Then, it was sonicated for 1 h and maintained at 80 °C for 5 days. Then, the graphite oxides (GOs) solution was washed 5 times with deionized (DI) water by centrifugation. The solution was dried in an oven at 60 °C for 24 h. The GOs (400 mg) were placed in dimethylformamide (DMF) (400 mL) and sonicated for 10 min. Zinc acetate dihydrate (18.4 g) was added to DMF (2 L). The GOs solution dispersed in DMF was added to the zinc acetate dihydrate solution. The reaction was carried out at 270 rpm for 5 h. After 2 h from the synthesis of QDs, the 4-AP (9.14 g) was added and reacted during 3 h. Subsequently, the mixed solutions were heated to 100 °C and maintained for 5 h in a constant temperature water bath, and then cooled down to room temperature. The solution was washed with ethanol and DI water 10 times with a centrifuge, and then dried in an oven at 80 °C for 4 days. The final ZGAs powder was obtained using filters.

### 3.2. Characterization of ZGAs and ZGQDs

The surface morphologies were observed using FE-SEM (Carl Zeiss Co. Ltd. MERLIN, Oberkochen, Germany) and TEM (Philips Tecnai G2 F20, Amsterdam, The Netherlands). The XRD measurement were performed by a Panalytical Empyrean using Cu Kα radiation. The FT-IR spectroscopy (JASCO FT/IR-6600, Tokyo, Japan) was used to characterize the chemical structures of the materials. The Raman spectra were collected by Raman equipment (WITec, Uim, Germany) with a 532 nm laser. UV-Vis absorption spectra were obtained through a jasco-V670 UV-Vis-NIR spectrometer. The PL emission was spectrally resolved using collection optics and a monochromator (SP-2150i, Acton, NJ, USA). The PL lifetimes were measured with a picosecond TRPL measurement system using a streak camera (C11200, Hamamatsu Photonics, Shizuoka, Japan) at room temperature with excitation source of 350 nm, 2nd harmonic of fs Ti:Sapphire laser.

### 3.3. Photoelectrochemical (PEC) Measurements

The photoanode films composed of ZGQDs and ZGAs were fabricated. The 0.25 g of ethyl cellulose with 5 mL of α-terpineol and 0.5 mL of butyl acetate were mixed. The mixture was stirred for 1 h and maintained for 12 h. The paste was prepared by dissolving the powder sample (0.65 g) into the mixture. The paste was uniformly deposited onto the top of the FTO glass using the conventional doctor blade method. Finally, the paste coated FTO glass was transferred into an oven for drying at 80 °C for 2 h. The electrochemical measurements were carried out using 0.5 M NaClO_4_ electrolyte (pH 6.9), a saturated Ag/AgCl reference electrode, and a Pt counter electrode with an electrochemical instrument (ZIVE SP1, WonATech, Seoul, Korea) with a solar simulator (1 kW solar simulator, Oriel, Stratford, CT, USA). To determine the active area, an imide tape with an aperture was used as a shadow mask. The whole surface of our photoanode samples (5 by 5 mm) was masked except the aperture area.

### 3.4. Photodegradation Experiment

Twenty-five milligrams of sample powders were dispersed into 50 mL of 0.01 mM Rhodamine B aqueous solution. The solutions were put in the dark condition for 30 min to provide adsorption/desorption equilibrium state. The solution was stirred under 1 sun (100 mW/cm^2^) irradiation. During the light irradiation, 1 mL of solution was extracted at intervals, and then centrifuged to obtain the decomposed solution. The concentration changes of the supernatant before and after photodegradation were measured using UV-Vis absorbance.

### 3.5. Fabrication of ZGA- and ZGQD-Based Photodetector and Measurements

Five milligrams of ZGAs and ZGQDs powders were dispersed into 10 mL of ethanol. The solutions of ZGAs and ZGQDs were spin-coated on the prepared Cr/Au (10 nm/100 nm) IDT patterned SiO_2_/Si substrate using the drop casting followed by annealing at 110 °C for 10 min under ambient condition. To obtain the electrical signal, a Keithely 4200 tester with the optical sources using a 150 W Xenon Lamp and monochromator was used.

## 4. Conclusions

In conclusion, ZnO-graphene core-shell QD aggregates were fabricated by solvothermal method and characterized for their structural and optical properties. The absorption of the ZGAs was increased in the wavelengths ranging from 350 to 750 nm by an enhanced strong light scattering effect. To investigate the effect of ZGAs on the device performance, the PEC cells and PDs were fabricated. The photocurrent density of PEC cells with ZGAs was reduced by about 3.25 times as compared with that of ZGQDs. Moreover, the photoresponsivity of the PDs with ZGAs was around twice as low as that of the PDs with ZGQDs. The degraded performance can be attributed to the increased carrier traps in the oxygen related functional groups of graphene shells and 4-AP, which is supported by a longer lifetime calculated from TRPL. The degradation of the device performance could be attributed to increased defect sites that limit the photoconversion activity. Consequently, modification of trap sites in ZGAs is essentially required for the improved photoconversion activity.

## Figures and Tables

**Table 1:** Photodetection performance of ZGAs and ZGQDs.

Sample	Rise Time (s)	Fall Time (s)	Darkcurrent (μA·cm^−2^)	Photocurrent (μA·cm^−2^)	Sensitivity	Responsivity (μA·mW^−1^)
ZGQDs	28.2	0.7	7.28	7.38	0.0137	3.85
ZGAs	29.9	0.8	8.16	8.2	0.005	1.54

## Supplementary Materials

The following are available online, Figure S1: Particle size distribution of ZGAs, Figure S2: SEM image of ZnO aggregates, Table S1: The table of PL lifetime of ZGAs and ZGQDs, Figure S3: Current-voltage (I-V) curves for the photodetector with ZGAs and ZGQDs under dark state.
