# Supplementary material for: Inhibition of Photoconversion Activity in Self-Assembled ZnO-Graphene Quantum Dots Aggregated by 4-Aminophenol Used as a Linker
Source: Molecules. 2020 Jun 17;25(12):2802. doi: 10.3390/molecules25122802 (PMC7355606; doi:10.3390/molecules25122802)
Supplement: Supplementary file 1 [file molecules-25-02802-s001.pdf]

Supporting Information

Inhibition of Photoconversion Activity in Self-Assembled ZnO-Graphene Quantum Dots Aggregated by 4-Aminophenol Used as a Linker

Kyu Seung Lee [1,†], Young Jae Park [2,†], Jaeho Shim [1], Guh-Hwan Lim [1], Sang-Youp Yim [3], Jin Won Seo [4], Jae Hyoung Ryu [2] and Dong Ick Son [1,5,*]

[1] Institute of Advanced Composite Materials, Korea Institute of Science and Technology, 92, Chudong-ro, Bongdong-eup, Wanju-gun, Jeollabuk-do 55324, Republic of Korea; kslee4268@gmail.com (K.S.L.); jshim0214@gmail.com (J.S.); gunanwow@gmail.com (G.-H.L.)

[2] Light Convergence Research Team, Korea Institute of Lighting and ICT, 370, Dongseo-ro, Iksan-si, Jeollabuk-do 54630, Republic of Korea; yjpark@kilt.re.kr (Y.J.P.); phynux@kilt.re.kr (J.H.R.)

[3] Advanced Photonics Research Institute, Gwangju Institute of Science and Technology, 123, Cheomdangwagi-ro, Buk-gu, Gwangju 61005, Republic of Korea; syim@gist.ac.kr

[4] Department of Materials Engineering, KU Leuven, 3001 Leuven, Belgium; maria.seo@kuleuven.be

[5] KIST School, Department of Nanomaterials and Nano Science, University of Science and Technology (UST), 217, Gajeong-ro, Yuseong-gu, Daejeon 34113, Republic of Korea

* Correspondence: eastwing33@kist.re.kr

† These authors contributed equally to this work

Academic Editor: Giuseppe Cirillo

Received: 19 May 2020; Accepted: 15 June 2020; Published: date

Table of contents

1. Particle size distribution of ZGAs.

2. SEM image of ZnO aggregates.

3. The table of PL lifetime of ZGAs and ZGQDs.

4. Current-voltage (I-V) curves for the photodetector with ZGAs and ZGQDs under dark state.

1. Particle size distribution of ZGAs

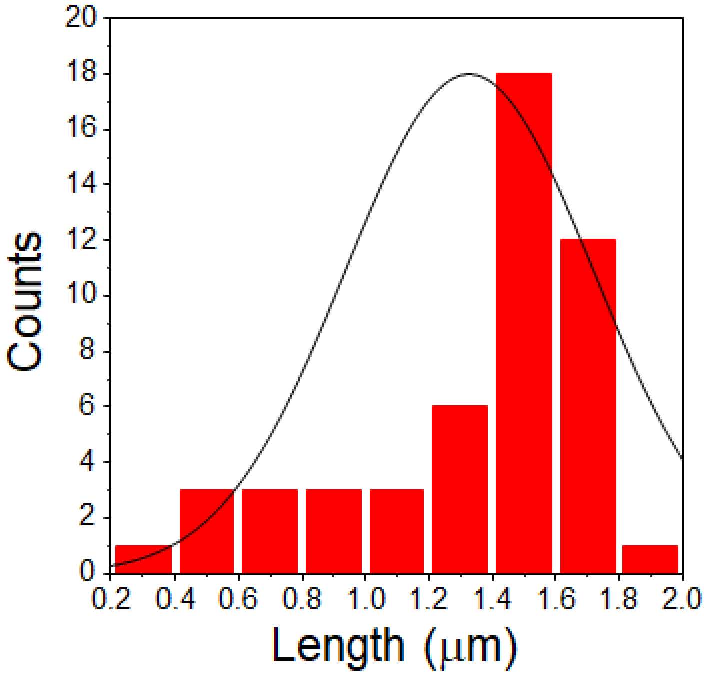

Figure S1. The particle size distribution of ZGAs was investigated with the SEM image of fig 1(e) using the image J software, which have distributed around ~1.5 μm.

2. SEM image of ZnO aggregates of irregular structures.

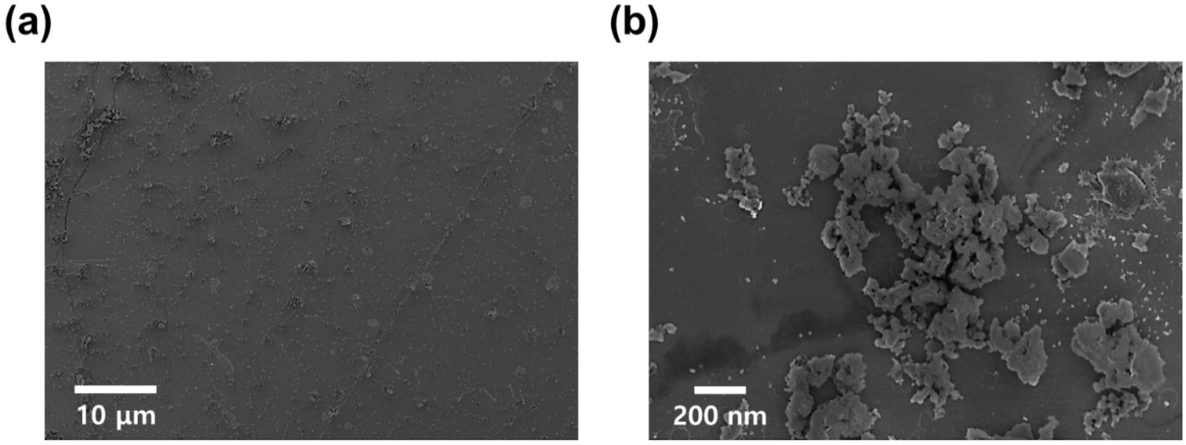

Figure S2. (**a** and **b**) The SEM images of ZnO aggregates were observed the irregular particle shape and nonuniformly structures.

3. PL lifetime of ZGAs and ZGQDs.

Table 1. PL lifetime of ZGAs and ZGQDs. Excitation: 350 nm, 2nd harmonic of fs Ti:Sapphire laser. The PL decay curves were fitted by a tri-exponential function to calculate the lifetime (τ_1, τ_2 and τ_3), signal amplitude (A_1, A_2, A_3), and average lifetime (τ_{avg}) of the samples. χ^2 is the reduced chi-squared value.

	τ_1	τ_2	τ_3	A_1	A_2	A_3	τ_{avg}	χ^2_{tri}	χ^2_{bi}
**ZGQDs**	0.11	2.36	9.96	0.985	0.011	0.004	2.72	1.94	5.29
**ZGAs**	0.11	2.33	10.3	0.804	0.126	0.07	7.37	1.73	2.60

* Unit: ns

If the plot of log versus time is not linear, this appears as a decay with several nearly linear segments each of which has a different slope. Therefore, we have fitted the TRPL data to a sum of three exponentials. Using tri-exponential fitting: $I(t) = A_1\exp(-t/\tau_1) + A_2\exp(-t/\tau_2) + A_3\exp(-t/\tau_3)$. Also, the reliability of TRPL data can be evaluated through the chi-square values differ according to the fitting method. To demonstrate a clearer difference, we present the chi-square values of bi-exponential and tri-exponential. The chi-square value through tri-exponential fitting is closer to 1 than the bi-exponential fitting respectively. Therefore, the tri-exponential fitting is appropriate for present TRPL data.

4. Current-voltage (I-V) curves for the photodetector with ZGAs and ZGQDs under dark state.

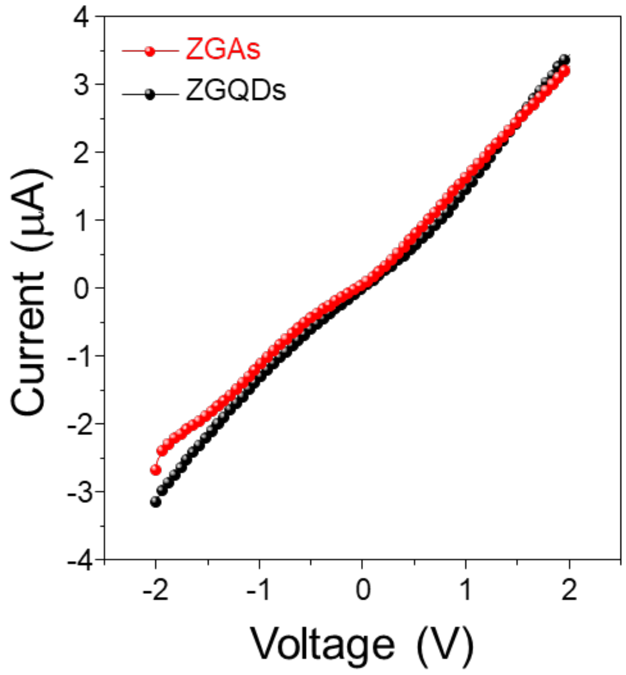

Figure S3. I-V curves for the photodetector with ZGAs and ZGQDs under dark state.
